# The Prevalence of Violence Against Women During Pregnancy and After Delivery in Saudi Arabia: A Cross-Sectional Study

**DOI:** 10.7759/cureus.26417

**Published:** 2022-06-29

**Authors:** Futun A Al-Khushayban, Maha K Alharbi, Muneera A Alsheha, Mansourah F Bedaiwi, Shahad S Alolayan, Renad I Aljasser, Afaf M Alanazi, Barah F Bedaiwi, Ftoun A Almuhaimeed, Fatimah K Almeathem

**Affiliations:** 1 Medicine, College of Medicine, Qassim University, Qassim, SAU; 2 Obstetrics and Gynecology, College of Medicine, Qassim University, Qassim, SAU

**Keywords:** delivery, pregnancy, women, violence, labor

## Abstract

Introduction

Violence against pregnant women has become a public health issue and a violation of human rights. The World Health Organization (WHO) defines violence as any act (physical or verbal) that causes physical or psychological harm. Obstetric violence committed by healthcare providers can lead to significant health consequences harming both mother and child. During pregnancy, violence is more frequent than some obstetric complications that are routinely recorded or screened. Therefore, this study aims to assess the prevalence of violence against women during pregnancy and labor, and postpartum.

Materials and methods

In this cross-sectional study, our study population consisted of women who have experienced pregnancy and labor in the Najd region. We used both face-to-face and online questionnaires that evaluated the knowledge and practice outcomes of women who have experienced violence during labor, in addition to the behavior of healthcare providers toward these women.

Results

In our analysis of demographic data, we found a significant association between age and having experienced violence before/during birth. Most women who experienced violence were between 25 and 45 years old (p=0.002). Furthermore, the history analysis revealed a significant association between follow-up regularity and violence experiences (p=0.010). Nursing students delivered most women (71%), and they did not provide information regarding the women’s rights or procedures. Of the respondents, 39.6% did not feel comfortable and were afraid of the healthcare providers’ words, phrases, or behaviors.

Conclusion

Our study concluded that many women experience violence committed by healthcare providers before, during, and after labor without realizing it. As a result of the ignorance of their rights, violence is more prevalent among these women. As a recommendation, to expand on the rights, women organizations should dedicate more efforts and throw campaigns to raise the awareness of violence among other women.

## Introduction

The World Health Organization (WHO) defines violence as any act (physical or verbal) that causes physical or psychological harm [1]. Violence against pregnant women has become a public health issue and a violation of human rights. Also, it is considered a hidden epidemic. Obstetric violence can lead to significant health consequences affecting both mother and child [2]. Violence against women during labor happens everywhere, regardless of culture and development. However, it is more prevalent in specific subgroups of the population. The prevalence of reported violence varies widely due to differences in the material, definitions, methodologies, and context [3,4].

Among victims of violence during pregnancy, there is a significant risk of miscarriage, placental abruption, preterm delivery, perinatal mortality, and low birth weight [5]. During labor, some women experience physical violence in the form of beatings by the nursing staff. They may also experience being forced to deliver on the floor and subjected to unnecessary cesarean sections [6,7]. This is known as obstetric violence and reveals itself through negligence, recklessness, omission, discrimination, and disrespectful acts by healthcare professionals. In Mexico, the national human rights commission and the government have included obstetric violence in the general law. Physical or verbal violence should be routinely assessed at obstetric clinics [8] because, despite its increased frequency and serious consequences, roughly two million newborns die within the first 24 hours of their lives every year. Pregnancy, childbirth, and the postpartum period account for more than 600,000 deaths among women each year [9].

Many factors contribute to violence during pregnancy, such as low educational levels, smoking, drug abuse, unwanted pregnancies, insufficient income, unemployment, forced marriage, and the physical and mental health of the couple [10]. As a result of a lack of awareness, some Saudi women justified violence. It is difficult for some women who have experienced violence to report it to the authorities [11].

The WHO and Pan American Health Organization have developed a series of information sheets that revealed the prevalence, patterns, consequences, risk factors, and strategies to prevent different forms of violence against women [1]. This study aimed to determine the prevalence of violence against women during pregnancy and after delivery, assess the awareness of obstetric violence among women, and evaluate the quality of healthcare assistance during childbirth in hospitals in the Najd region of Saudi Arabia.

## Materials and methods

Study design, area, population, and sampling 

This is a cross-sectional study carried out in the Najd region of Saudi Arabia. We obtained ethical approval from the Committee of Research Ethics, Deanship of Scientific Research, Qassim University, Buraydah, Saudi Arabia (approval number 190104). We distributed the questionnaire online and at any female social gatherings. All women who gave birth were included. Women participated conveniently, and their consent was taken preceding the questionnaire. They agreed to participate in the study and were informed of the purpose, confidentiality rights, and right to withdraw at any time without any obligation to the study team.

Tools, size, and selection of sample

We received a total of 753 women. Our minimum sample size was 350 calculated using the following formula:

$N=\frac{z^{2}{}pq}{d^{2}}$

$N=\frac{1.96^{2}{}(.35)(1-.35)}{0.05^{2}}$ = 350

p is the expected prevalence, which is 35% based on previously published studies; q=1-p; d is the absolute error or precision, which is 5% (0.05); z is the standard normal variant at 5% type 1 error (p<0.05), which is 1.96. The total population of Riyadh is about five million, and that of Qassim is about 1.5 million.

A validated questionnaire [12] was distributed both online and face-to-face. The questionnaire consisted of three sections. The first section included the demographic data of the participants, including age, education, marital status, work, and monthly household income. The second section included obstetric history, which includes the number of pregnancies, abortions, fetal outcome, follow-up, privacy, and forced supine position. In addition to pre-labor data, data on labor time, companion, rules notification, help, checking fetal pulse, intravenous treatment, and vaginal examination were also collected. The third section included data of delivery, such as method, place, help, doctor, position, drinks offered, verbal violence, questions, induction, cuts, and wounds. In addition to postdelivery data, data about holding the child, breastfeeding, mothers’ satisfaction, maternity leave, and cleaning up were included.

Statistical analysis

Data analyses were performed using SPSS Statistics for Windows version 26.0 (IBM Corp., Armonk, NY, USA). Descriptive statistics were summarized using numbers, percentages, means, and standard deviations. The chi-square test was used to determine if there is a significant relationship between two nominal (categorical) variables. There are several factors to consider, such as marital status (married, divorced, or widowed), educational level (educated or not educated), monthly household income, the type of hospitals (private or public), the number of deliveries, the type of violence (verbally or physically), and the position of the delivery. P-value < 0.05 was considered statistically significant.

## Results

Demographic characteristics of women

A total of 753 women participated in this study, of whom most (84.1%) were between the ages of 25 and 45, with just 7% of them being over 45 years. Based on their educational level, it was found that 169 (22.4%) had stopped at preparatory school, while the majority of 534 (70.9%) had completed secondary school. In the high education group, 15 (2%) participants received bachelor’s degrees, six (0.8%) earned master’s degrees, and two (0.3%) received doctoral degrees. The majority of women (n=713 (94.7%)), were married, 32 (4.2%) were divorced, and eight (1.1%) were widows. As regards work, 437 (58%) respondents reported being employed, whereas 316 (42%) reported being housewives. The financial status of working women revealed that 343 (45.6%) had an income exceeding 10,000 RS, 308 (40.9%) between 5,000 and 10,000 RS, and 102 (13.5%) only had an income below 5,000 RS (Table 1).

**Table 1:** Demographic characteristics of women (n=753)

Variables		Frequency	Percentage
Age	Less than 18 years	1	0.1
	18-25 years	66	8.8
	25-30 years	357	47.4
	30-45 years	276	36.7
	More than 45 years	53	7
Education	Uneducated	12	1.6
	Primary school	15	2
	Preparatory school	169	22.4
	Secondary school	534	70.9
	Bachelor’s degree	15	2
	Master’s degree	6	0.8
	PhD	2	0.3
Marital status	Married	713	94.7
	Divorced	32	4.2
	Widow	8	1.1
Working		437	58
Not working		316	42
Income	Less than 5,000	102	13.5
	Between 5,000 and 10,000	308	40.9
	More than 10,000	343	45.6

Pre-labor period

Based on their pre-labor data, 618 (82.1%) were born full-term with normal labor. A total of 452 (60%) did not have the right to choose a companion, and 462 (60.9%) were forced into a supine position, while only 56 (31.0%) of the participants were not forced into a specific position during labor. Out of 753 participants, 512 (68%) were not informed of the rules to having a companion during childbirth. The study found that 365 (48.5%) respondents had not received helpful instruction, with a significant association (p=0.060). Almost all (90%) of the 753 women were examined vaginally during labor, of which 370 (53.5%) were examined by nursing students, and only 117 (17%) were examined by doctors (Table 2).

**Table 2:** Pre-labor period (n=753)

		Frequency	Percentage
In which month did the labor begin?	Before 28 weeks	16	2.1
	Before 34 weeks	13	1.7
	Before 36 weeks	106	14.1
	36-40 weeks	618	82.1
Did you have the right to choose a companion with you during labor and childbirth?	Yes	250	33.2
	No	452	60
	Not sure	51	6.8
If yes, who was he/she?	Husband	137	55
	Mother	66	26
	Sister	42	17
	Friend	5	2
	Total	250	100
Have you been notified of the accompanying rules?	Yes	174	23.1
	No	512	68
	Not sure	67	8.9
What kind of help did you receive?	Breathing exercises	52	34
	Massages	60	39
	Walking	32	21
	Shower	5	3
	Others	4	2
During labor, did the medical staff check the fetus’s pulse?	Yes	654	86.9
	No	48	6.4
	Not sure	51	6.8
Did they put intravenous treatment when you entered?	Yes	523	69.5
	No	130	17.3
	Not sure	100	13.3
Have you been instructed not to drink or eat during labor?	Yes	321	42.6
	No	465	48.5
	Not sure	67	8.9
Have you been examined through your vagina during labor?	Yes	678	90
	No	58	7.7
	Not sure	17	2.3
If yes, who did the vaginal examination?	Nurse	193	28
	Doctor	117	17
	Nursing student	370	53.5
	Medical student	2	0.5
	Other	3	0.5

**Figure 1:**
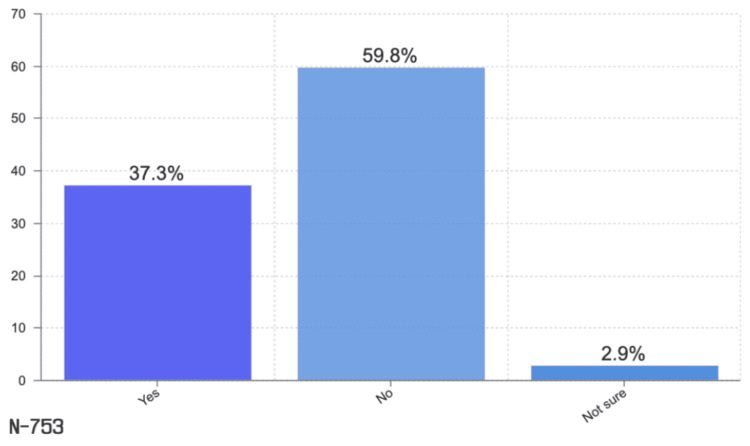
Pre-labor help from the hospital medical staff

Based on our analysis of pre-labor help provided by hospital medical staff to pregnant women to relieve cramps or pain, 59.8% answered no help, 37.3% answered some help, and 2.9% were not sure if any help was provided (Figure 1).

The analysis of some incidents related to pregnant women (history and pre-labor) found a significant association between age and violence (p=0.002); educational level also showed a significant association with violence (p=0.000). There was a significant association between the number of pregnancies and violence exposure among participants (p=0.010). Of the participants, 258 (34%) had one miscarriage, and 39 (5.2%) had a dead baby after delivery, with a significant association (p=0.040). In addition, there is a significant association between the experience of violence and regularity of follow-up, feeling of privacy, and forced supine position (Table 3).

**Table 3:** Factors (history and pre-labor) associated with violence § P-values were calculated using the chi-square test. ** Significant at p<0.05 level.

Demographic data	Yes (frequency (%))	No (frequency (%))	_χ_2	p-value§
Age (years)				
>18	1 (0.4)	0 (0)	24.660a	0.002**
18-25	17 (7.4)	40 (8.2)		
25-30	119 (51.7)	232 (47.3)		
30-45	80 (34.8)	181 (36.9)		
>45	13 (5.7)	37 (7.5)		
Level of education				
Uneducated	2 (0.8)	10 (2.04)	12.997a	0.000
Primary education + intermediate education	64 (13.06)	112 (22.9)		
High school education + higher education	164 (71.3)	368 (75.1)		
Number of pregnancies (have you had an accident/violence before/during birth?)				
One-time pregnancy	47 (20.4)	109 (22.2)	31.943a	0.010**
Two-time pregnancy	109 (47.4)	256 (52.2)		
Three-time pregnancy	27 (11.7)	50 (10.2.1)		
Four-time pregnancy	25 (10.9)	25 (5.1)		
Five-time pregnancy	9 (3.9)	20 (4.08)		
Six-time pregnancy	4 (1.7)	13 (2.7)		
Seven-time pregnancy	7 (3.04)	10 (2.04)		
Eight-time pregnancy	2 (0.9)	5 (1.02)		
Nine-time pregnancy	0 (0)	2 (0.4)		
Fetal outcome (did any of your children die after delivery?)				
Women who experienced violence during/before birth	19 (8.3)	211 (91.7)	6.448a	0.040**
Women who did not experience violence during/before birth	19 (3.9)	471 (96.1)		
Women who are not sure	1 (0.4)	32 (6.5)		
Regular follow-up (have you been to pregnancy follow-up regularly?)				
Women who experienced violence during/before birth	214 (93.04)	16 (6.95)	9.181a	0.010**
Women who did not experience violence during/before birth	456 (93.06)	34 (6.9)		
Women who are not sure	26 (78.8)	7 (21.2)		
Feeling privacy (did you feel privacy during labor?)				
Women who experienced violence during/before birth	77 (33.5)	149 (64.8)	127.276a	0.000
Women who did not experience violence during/before birth	348 (71.02)	109 (22.2)		
Women who are not sure	19 (57.6)	10 (30.3)		
Forced supine position (have you been forced to lay on your back during labor?)				
Women who experienced violence during/before birth	214 (93.04)	16 (6.95)	9.181a	0.010**
Women who did not experience violence during/before birth	456 (93.06)	34 (6.9)		
Women who are not sure	26 (78.8)	7 (21.2)		

During labor

When we asked the participants about their delivery, 720 (95.6%) were in the delivery room, and 737 (95.6%) had medical assistance, but nursing students delivered most of the women (n=526 (71%)). A total of 737 (97.9%) women delivered their babies while in the supine position. Of the participants, 514 (68.3%) underwent episiotomy, and 281 (46%) received anesthesia, while 190 (31%) did not. Since 143 (23%) respondents were uncertain about the use of anesthesia, we asked if they felt pain while their perineums were sewn; 336 (51%) responded in the affirmative and 243 (37%) in the negative (Table 4).

**Table 4:** During labor (n=753)

		Frequency	Percentage
How was the birth delivered (except for miscarriage)?	Normal	574	76.2
	Cesarean	179	23.8
Where did you give birth?	Delivery room	720	95.6
	Before delivery room	21	2.8
	Other	12	1.6
Was the birth with the help of medical staff?	Yes	737	95.6
	No	16	2.8
If yes, who did the delivery?	Nurse	76	10
	Doctor	127	17
	Nursing student	526	71
	Medical student	3	1
In which position was the delivery done?	Lying on back	737	97.9
	Lying on side	2	0.3
	Squatting	13	1.7
	Standing	1	0.1
	Sitting	0	0
Did the medical staff offer drinking liquid (water, juice, and/or tea) during childbirth?	Yes	77	10.2
	No	644	85.5
	Not sure	32	4.2
Have you been notified/advised of other positions to help you during childbirth?	Yes	196	26
	No	524	69.6
	Not sure	33	4.4
Have you ever felt uncomfortable and afraid of any word/phrase/behavior mentioned or done by the medical staff during labor and delivery?	Yes	298	39.6
	No	407	54.1
	Not sure	48	6.4
Did you ask any questions while you were in labor?	Yes	474	62.9
	No	226	30
	Not sure	53	7
If yes, have your questions been answered?	Yes	350	65
	No	140	26
	Not sure	50	9
Have labor induction been used?	Yes	380	50.5
	No	312	41.4
	Not sure	61	8.1
Has your belly been pressed to facilitate the delivery process?	Yes	328	43.6
	No	348	46.2
	Not sure	77	10.2
Was the perineum cut during delivery?	Yes	514	68.3
	No	200	26.6
	Not sure	39	5.2
If yes, did you receive anesthesia while the perineum was cut?	Yes	281	46
	No	190	31
	Not sure	143	23
	Total	614	100
Have you felt pain while the perineum was sewn even with anesthesia?	Yes	336	51
	No	243	37
	Not sure	76	12
	Total	655	100

**Figure 2:**
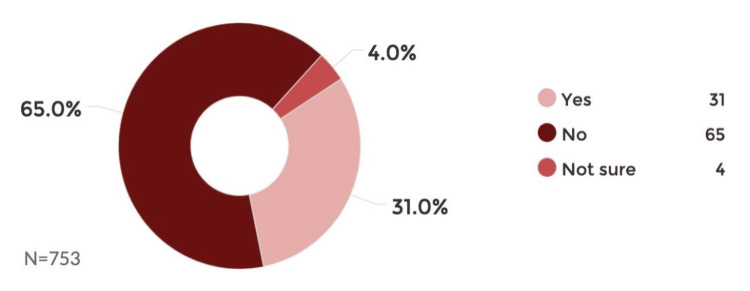
Prevalence of accidents/violence before or during delivery

According to our assessment of the prevalence of violence experienced by our participants before or during labor, 31% of the 753 women experienced violence before or during childbirth, 65% had no accident in their opinion, and 4% were not sure (Figure 2).

Post-labor period

When we asked the mothers whether they held their newborns after delivery, 356 (47.3%) did not hold them in the delivery room, while 378 (50.2%) did. Of the women, 332 (44.1%) had not been notified of the need to breastfeed during the first hour following delivery. A total of 294 (39%) women reported feeling unsatisfied or unsupported by the medical staff during labor and childbirth, and 23 (3.1%) women were unsure whether they felt supported and satisfied by the medical staff. Meanwhile, 230 (30.5%) respondents did not have the right to take maternity leave during childbirth, while 312 (41.2%) did (Table 5).

**Table 5:** Post-labor period (n=753)

		Frequency	Percentage
Did you hold your child in the delivery room?	Yes	378	50.2
	No	356	47.3
	Not sure	19	2.5
Have you been notified of the need to breastfeed your baby during the first hour after birth?	Yes	398	52.9
	No	332	44.1
	Not sure	23	3.1
Have you felt satisfied/supported by the medical staff during labor and childbirth?	Yes	417	55.4
	No	294	39
	Not sure	42	5.6
Have you had the choice to take maternity leave during your childbirth?	Yes	312	41.4
	No	230	30.5
	N/A	211	28
Have you been washed/cleaned topically?	Yes	519	68.9
	No	128	17
	Not sure	106	14.1

## Discussion

Violence against women is becoming one of the most common social, public, human rights, and health concerns [13]. Violence against women may occur during pregnancy, according to global research. Based on a multicountry study, up to 28% of pregnant women have experienced physical violence [14]. In addition, there is evidence that violence may negatively impact the health of women and infants, resulting in delays in seeking antenatal care, miscarriage, preterm delivery, and low birth weight [6]. During pregnancy, violence is more frequent than some obstetric complications, because women are not fully aware of their rights during this critical and valuable time. In this study, most of the respondents’ ages (84.1%) ranged from 25 to 40 years, and 70.9% had a secondary educational level. In addition, 35% did not feel privacy, 60% did not have the right to choose a companion, and 68% had not been notified about the rules. There are several types of violence, such as verbal, emotional, and physical, as well as even obscure laws and rights intended to protect women [14]. We estimated that 450 (59.8%) participants in our study did not receive any medication to relieve pain nor were provided reassurance. According to our study, some answers reflected the percentage of clueless women. During childbirth, 33 (4.4%) participants are unaware of other positions available to help them; 7%-23% of women did not receive any information about their rights, including asking questions and trying the essential procedures that could be helpful. Although most women have had multiple pregnancies, they report violence only during one of their pregnancies. It is necessary to conduct further research to gain a deeper understanding of this issue and develop appropriate prevention strategies [15].

After analyzing our results, we found that many women are victims of various types of abuse during the delivery process. It was reported that medical and premedical staff at health institutions treated them disrespectfully. The effects of this reality were felt in several countries throughout the world. Abusing these women violates their right to quality healthcare, and it also puts their physical and mental integrity at risk at a time of extreme vulnerability [16]. Several studies have found an association between violence and poor pregnancy outcomes. For example, women who had experienced abuse during pregnancy are more likely to register for late prenatal care, experience premature labor, and give birth to low-weight children [17]. Besides the physical trauma caused by assaults, such as punches or slaps, continuous stress also has another consequence. It has been shown that continuous stress has a negative impact on the perinatal outcome through changes in individual behavior or physiological responses. Individuals can engage in various types of behaviors, which can interfere with their ability to maintain a healthy nutritional status, rest, or receive medical care [18]. We found that 462 (61.4%) participants had been forced into a supine position without receiving intravenous treatment. Contrary to WHO recommendations, women should give birth in the position they are most comfortable in [19]. In addition, 90% of the participants underwent vaginal examination (53.5%) by nursing students during the pre-labor period, which is considered physical abuse during pregnancy. Compared to the Multi-country Study on Women’s Health and Domestic Violence against Women, sponsored by the World Health Organization, between 2000 and 2003, this result is very similar [14].

During all stages of pregnancy, pregnant women are subjected to multiple forms of violence, but despite this, health-seeking behavior is minimal following the violent assault. Their results reflect poor awareness of gender-based violence among healthcare providers [20]. When testing multiple factors associated with having an accident or violence before birth, there was a significant association between most labor factors and having an accident or violence before or during birth. The incidence of perinatal and neonatal mortality is higher in women who have suffered physical violence during pregnancy than in women who did not suffer such violence during pregnancy [21]. We found that 67% of women who experienced violence during labor delivered in public hospitals. While most of them (95.6%) were in the delivery room, 39.6% reported feeling uncomfortable or afraid. Multicountry studies of the prevalence of different abuse types among women seeking antenatal care suggest that these women are likely to have a history of abuse. The different forms of abuse prevailed and varied significantly between the participating countries, with or without adjusting for age, education, and gestational length [22]. In contrast, estimates of the prevalence of past and present violence and abuse in pregnant women vary greatly and may be difficult to compare. This is because they differ regarding the type of abuse assessed, the time of occurrence, and the perpetrator [23]. They added that methodological factors such as the study design, measuring instrument, and population studied could influence results. Our study found that 187 (63%) women experienced verbal violence and 111 (37%) women experienced physical violence but never reported these incidents. These results were consistent with a retrospective, cross-sectional study in Bangladesh [24]. Furthermore, the authors linked violence against women to postpartum depression, which can be life-threatening in some cases. It is important to consider all of these factors as indicators that can alert healthcare providers. This will enable them to develop or improve guidelines and plans of action to help pregnant women who have been exposed to violence [3].

Limitations

The obstetric field faces many challenges, particularly in Saudi Arabia. Our study represents a region, not the whole country. The results are based on a self-administered survey; hence, reporting bias cannot be eliminated. The generalization of this study’s results should be made carefully.

## Conclusions

Pregnancy on its own is a very stressful event for the mother’s body and mental health. Healthcare providers should work better for pregnant women to eliminate the unnecessary physiological and psychological suffering during and after giving birth. Some of the practices developed in the daily routine of obstetric services are inconsistent with public health recommendations. Our results suggest that unnecessary interventions may result in physical or psychological abuse and serious problems. Therefore, obstetric healthcare providers, especially nurses and doctors, must seek continued professional development to develop their care activities according to public health policies. We concluded that screening for violence during prenatal care must be further studied and applied since the gestation period is sensitive and critical to a mother’s future health. We must encourage women to seek help if they are facing any type of violence. We recommend health organizations in the region to throw campaigns to enhance the awareness of women in the community about their rights during pregnancy and labor, and postpartum.
